# Is There Any Association Between Periodontitis and Prostatic Alterations? A Systematic Review

**DOI:** 10.1002/pros.70029

**Published:** 2025-08-10

**Authors:** Beatriz Rodrigues Risuenho Peinado, Rayssa Maitê Farias Nazário, Deborah Ribeiro Frazão, Yago Gecy de Sousa Né, Leonardo Oliveira Bittencourt, Nathália Carolina Fernandes Fagundes, Caio Melo Mesquita, Cassiano Kuchenbecker Rösing, Renata Duarte de Souza‐Rodrigues, Luiz Renato Paranhos, Lucianne Cople Maia, Rafael Rodrigues Lima

**Affiliations:** ^1^ Laboratory of Functional and Structural Biology, Institute of Biological Sciences Federal University of Pará Belém Brazil; ^2^ Division of Preventive and Community Dentistry, School of Dentistry Federal University of Uberlandia Minas Gerais Brazil; ^3^ Department of Periodontology, Faculty of Dentistry Federal University of Rio Grande do Sul Porto Alegre Brazil; ^4^ Department of Pediatric Dentistry and Orthodontics, School of Dentistry Federal University of Rio de Janeiro Rio de Janeiro Brazil

**Keywords:** benign prostatic hyperplasia, periodontitis, prostate neoplasms, prostatitis, systematic reviews

## Abstract

**Background:**

The prostate plays a crucial role in male reproduction but is susceptible to diseases such as prostate cancer. Periodontitis, as an inflammatory disease, has the potential to modulate systemic conditions. This systematic review aimed to evaluate the association between periodontitis and prostatic alterations.

**Methods:**

The review was conducted in accordance with the Preferred Reporting Items for Systematic Reviews and Meta‐Analyzes (PRISMA) guidelines and registered in the PROSPERO database (CRD42024614333). Observational studies comparing the presence of periodontitis in men with and without prostatic alterations were included. The search strategy was applied to databases such as PubMed, Scopus, Embase, Web of Science and Lilacs, as well as gray literature (OpenGrey and Google Scholar). The selection of studies and data extraction were carried out independently by two reviewers. Methodological quality was assessed using the tools of the Joanna Briggs Institute, and confounding factors were analyzed using multivariate models, where applicable.

**Results:**

A total of 769 references were identified, and 14 studies were included. Most studies indicated a significant association between periodontitis and prostate cancer, with a higher risk in patients with periodontal disease. Associations with BPH and chronic prostatitis were also observed in fewer studies. However, methodological limitations, such as inadequate control of confounding factors (e.g. smoking, genetics and age), heterogeneity in diagnostic criteria and reliance on self‐reported data, increased the risk of bias. Many studies did not adequately adjust for confounding factors, compromising the robustness of the evidence.

**Conclusion:**

Thus, the findings suggest a potential association between periodontitis and prostatic alterations, especially prostate cancer.

## 1 Introduction

The prostate gland is situated inferior to the bladder and anterior to the rectum inside the male anatomy, constituting an integral component of the reproductive system. Its main role is to produce prostatic fluid, which, among other functions, accompanies and protects the male reproductive cells and is essential in the reproduction process. This organ is susceptible to several aggressive pathological diseases, including benign prostatic hyperplasia, prostatitis, and prostate cancer, which is recognized as one of the most prevalent forms of cancer globally. These disorders may arise due to hormone imbalances, genetic predisposition, and age‐related factors. Environmental risk factors include smoking, excessive alcohol consumption, diet, and infections [1, 2, 3].

One of the most widely used ways of initially diagnosing prostate diseases is by measuring blood levels of PSA, a specific antigen produced almost exclusively by prostate epithelial cells, whose primary function is to liquefy seminal fluid. Elevated levels over 10.0 ng/mL in the bloodstream serve as a potential indicator of prostate issues, particularly cancer. However, it is imperative to supplement this finding with additional diagnostic procedures, including rectal examination and biopsy, to establish a conclusive diagnosis [4, 5, 6].

Although diseases of the oral cavity are commonly considered as specific and isolated conditions during clinical routine, there is already substantial data showing that oral health is directly associated with other chronic disorders. Periodontal disease specifically has been associated with several of these pathologies, such as diabetes, rheumatoid arthritis, Alzheimer's disease as well as respiratory, cardiovascular, and neurological diseases [7, 8, 9, 10, 11, 12, 13]. The association between oral and systemic health is increasingly recognized, with periodontal disease linked to various systemic conditions such as diabetes, cardiovascular disease, and Alzheimer's disease [14, 15]. This connection is mediated by multiple mechanisms, including microbial dysbiosis, altered host immune responses, and systemic inflammation [15, 16].

Periodontitis is described as an inflammatory disease triggered by bacteria from oral biofilms, affecting the supporting tissues of the teeth, that is, the gingiva, periodontal ligament, and alveolar bone, leading to tooth loss as it progresses [15]. Environmental factors, such as smoking, alcoholism, diet, and endogenous factors, such as genetic susceptibility and psychological stress can modulate the course and severity of this disease. This progression can be quite aggressive, given that inflammatory response and consequent tissue destruction induce a positive feedback cycle of protein degradation, inflammation, and pathogen enrichment [15, 17, 18].

Recent studies reveal a strong link between periodontitis progression and oxidative stress, in which the host immune response promotes pro‐inflammatory cytokines, matrix metalloproteinases, and reactive oxygen species (ROS) that drive the tissue destruction seen in periodontal disease [18]. The upregulation of ROS not only advances periodontitis but also contributes to other inflammatory conditions by increasing oxidative stress. Consequently, inflammatory diseases that elevate systemic oxidative stress may reinforce each other cyclically, sustaining a redox imbalance that perpetuates disease progression [18, 19].

In parallel, oxidative stress is also a key factor in the pathogenesis of benign prostatic hyperplasia, prostate cancer, and chronic prostatitis, where it leads to epigenetic alterations, DNA damage, and cell transformations that can be hyperplastic or precancerous. This stress further intensifies the inflammatory cascade observed in prostatitis [20, 21]. Additionally, periodontitis‐related bacteria may translocate systemically, triggering pro‐inflammatory molecule release and chronic immune activation, contributing to a persistent systemic inflammatory state. Such inflammation has been linked to greater susceptibility to systemic diseases and suggests a potential bidirectional relationship [22, 23]. Thus, this pro‐inflammatory response could elucidate a bidirectional link between periodontitis and other chronic diseases, such as prostate diseases.

Ultimately, the literature has shown a surprising association between prostate pathologies and periodontitis. DNA matching that of periodontal pathogens, typically found in subgingival calculus or biofilm samples, has been identified in the prostate fluid of affected individuals [24]. This finding suggests a potential for these pathogens to disseminate through the bloodstream to the prostate's intraepithelial tissues, potentially causing histomorphological changes in the organ [25].

Therefore, due to the significant number of primary studies exploring a possible association between periodontitis and prostate diseases, this systematic review aims to compile and evaluate the current scientific evidence on this topic.

## 2 Materials and Methods

### 2.1 Protocol and Registration

The protocol was reported according to the Preferred Reporting Items for Systematic Review and Meta‐Analysis Protocols (PRISMA‐P) [26] and is accessible through the International prospective register of systematic reviews (PROSPERO) database under the number (CRD42024614333). This systematic review is reported following the Preferred Reporting Items for Systematic Reviews and Meta‐Analyzes (PRISMA) guidelines [27] and was conducted according to the Joanna Briggs Institute (JBI) Manual [28].

### 2.2 Selection Criteria

The purpose of this review was to determine whether there is an association between prostate alteration and periodontitis in men adult patients. The eligibility criteria were defined using the PECO strategy, where “P” represents the patients (Men), “E” represents the exposure (Presence of prostatic alterations), “C” group of comparison (Absence of prostatic alterations), and “O” represents the outcome (Periodontitis).

The inclusion criteria for selection of articles were as follows: (1) observational studies as case‐control, cross‐sectional or cohort studies being conducted in human adult men, and (2) studies which the focus was the comparison of participants with and without any type of prostate alteration. There were no restrictions on the language or year of publication. We excluded all articles that did not follow the PECO scheme and the following type of articles and documents: Case reports, reviews, meta‐analyzes, descriptive studies, opinion articles, letters to the editor, technical articles, editorials, personal opinions, books, and book chapters, studies in animals, children or adolescents and in vitro studies.

### 2.3 Search Strategy

To avoid selection bias, two authors (BRRP and RMFN) independently conducted the searches. A search strategy composed of MeSH and free terms to conduct a systematic search was utilized for the following online databases: PubMed, Web of Science, Embase, Scopus, Lilacs, and gray literature (Google Scholar and Open Gray). “Adult,” “Prostatic disease,” and “Periodontitis” were the primary terms used to construct search strategies. Several combinations of descriptors were conducted using the Boolean operators 'AND' and 'OR', considering the syntax rules of each database (Supporting Information S1). Searches were regularly updated in all databases until November 2024. The retrieved studies were imported into the EndNote X9TM software (Clarivate Analytics, Philadelphia, USA), where duplicates were automatically removed by the software itself, and the remaining duplicates were removed manually.

### 2.4 Process of Selecting Studies

Before selecting the studies, two examiners conducted a calibration exercise in which they examined the eligibility criteria and applied them to 20% of the retrieved studies to determine inter‐examiner agreement. Once a sufficient level of agreement (Kappa ≥ 0.81) was attained, the selection procedure initiated.

Based on the titles and abstracts, the studies were excluded or selected for full reading by two reviewers (BRRP and RMFN). In case of disagreements between evaluators, a third evaluator was consulted (DRF). After the initial phase of selection, the complete texts of potentially eligible studies were obtained and carefully reviewed. Three studies could not be located by traditional means, so a bibliographic request was made to the library's database (COMUT), and an email was sent to the corresponding authors to obtain the texts.

### 2.5 Data Extraction

After the complete reading of the included studies, two reviewers (BRRP and RMFN) independently extracted the following data: Authors, year, country, study design, main characteristics of the participants (origin, sample size and age), diagnosis of prostatic alterations, diagnosis of periodontitis and results. When there was disagreement or doubts about the included data, a third reviewer (DRF) reviewed the questions.

### 2.6 Assessment of Methodological Quality/Risk of Bias of Eligible Studies

Two reviewers (CMM and LRP) independently assessed the risk of bias in the selected studies using specific tools for each observational study design. For cross‐sectional studies, the tool “JBI Critical Appraisal Tools for use in JBI Systematic Reviews ‐ Checklist for Analytical Cross‐sectional Studies” [28] was used. For cohort studies, the “JBI Critical Appraisal Tools for use in JBI Systematic Reviews ‐ Checklist for Cohort Studies” tool was used [28]. Any disagreements between the reviewers were resolved by discussion and consultation with a third reviewer (RRL).

### 2.7 Evaluation of Control Statements for Potential Confounders and Bias Consideration

This evaluation was adapted from the methodology reported by Hemkens et al. (2018) [29] Initially, all eligible studies were screened to determine whether they performed multivariate analysis for controlling potential confounders (i.e., analysis of covariance—ANCOVA, multivariate analysis of covariance—MANCOVA, multivariate linear regression, multivariate logistic regression, multivariate Cox proportional hazard regression models). Studies that did not perform multivariate analysis were excluded from further consideration.

The remaining studies underwent a critical appraisal by two independent reviewers (CMM and LRP), with disagreements resolved by a third reviewer (RRL). The Abstract and Discussion sections of each eligible study were reviewed and evaluated using six predefined questions: (1) “Is the term “confounding” mentioned in Abstract or Discussion?”; (2) “Is the term “bias” used in Abstract or Discussion?”; (3) “Is any specific mention about non‐adjusted variables in Abstract or Discussion?”; (4) “Is there any mention about confounders affecting results in Abstract or Discussion?”; (5) “Is there any statement about the need for caution in interpretating the results?”; and (6) “Does Conclusion include any limitation about confounders?”. Table 1 shows the criteria for assessing control statements for potential confounding factors and consideration of bias.

**Table 1:** Evaluation of control statements for possible confounders and bias consideration.

Section	Question	Possible answers with explanation	*N* (%)
Abstract and discussion	Is the term “confounding” mentioned in abstract or discussion?	**Specific:** if authors used the exact term “confounding.”	7 (58.33%)
		**Alluded:** if authors used a similar term or phrase.	5 (41.67%)
		**No:** if the authors used neither the exact nor similar term.	0
	Is the term “bias” used in abstract or discussion?	**Yes:** if authors used the term “bias.”	5 (41.67%)
		**No:** if authors did not use this term.	7 (58.33%)
	Is any specific mention about non‐adjusted variables in abstract or discussion?	**Yes:** if there was specific mention about non‐adjusted variables with no reasons presented.	2 (16.67%)
		**Not measured:** if there was specific mention about non‐adjusted variable not being measured.	10 (83.33%)
		**Other reasons:** if there was specific mention about non‐adjust variables and with plausible reasons for not adjusting them.	0
		**No reasons:** if there was specific mention about non‐adjusted variables and with implausible reasons for not adjusting them.	0
		**No:** if there was no mention about any non‐adjusted variable.	0
	Is there any mention about confounders affecting results in abstract or discussion?	**Likely:** if authors used terms such as “likely” or convincing statements that confounders were not controlled.	2 (16.67%)
		**Possibly:** if authors used terms such as “possibly” or unsure statements that confounders were or were not controlled.	8 (66.66%)
		**Unlikely:** if authors used terms such as “unlikely” or convincing statements that confounders were controlled.	2 (16.67%)
		**No mention:** if there was no mention about this possibility.	0
	Is there any statement about the need for caution in interpretating the results?	**Yes:** if there was explicit mention about the need for caution in interpretating the results obtained in the study.	9 (75%)
		**No mention:** if there was no mention about this need for caution.	3 (25%)
Conclusion	Does conclusion include any limitation about confounders?	**Yes:** if there was a mention about this limitation.	5 (41.67%)
		**No:** if there was no mention about this limitation.	7 (58.33%)

### 2.8 Assessment of Confounding Factors

This assessment was modified from the original approach described by Wallach et al. (2020) [30]. The critically appraised studies had their Methods and Results sections read and evaluated by two independent reviewers (CMM and LRP), with any conflicts adjudicated by a third reviewer (RRL). This step facilitated the identification of variables and confounding domains present in each assessed study. The identified variables were classified into four categories: adjustment, stratification, matching, and restriction variables. Sex was not included as a variable in the assessment because the population was already restricted to males considering the outcome of prostatic alterations.

Adjustment variables were those used to adjust potential confounders in multivariate analyzes. They were also categorized into categorical (those measured qualitatively), continuous (quantitatively), categorical or continuous (both measurement methods were used), and unclear (measurement not clearly reported). Stratification variables were categorized into sampling (those used to create strata in the sampling process) and analysis (used to stratify the data analysis). Matching variables were those used to pair known characteristics of study participants, and they were categorical (measured qualitatively) or continuous (quantitatively). Restriction variables were those used to restrict the eligibility criteria in sampling (to limit the selection of participants) or analysis (to analyze data from specific groups of participants).

The proportion of adjustment was calculated using the total of different variables identified across the selected studies and the number of adjustment variables identified. The analysis of the calculated proportion was based on Cohen's Kappa thresholds and was classified as: very poor (<20%), poor (21%–40%), average (41%–60%), good (61%–80%), or very good (>80%).

## 3 Results

### 3.1 Study Selection

**Figure 1:**
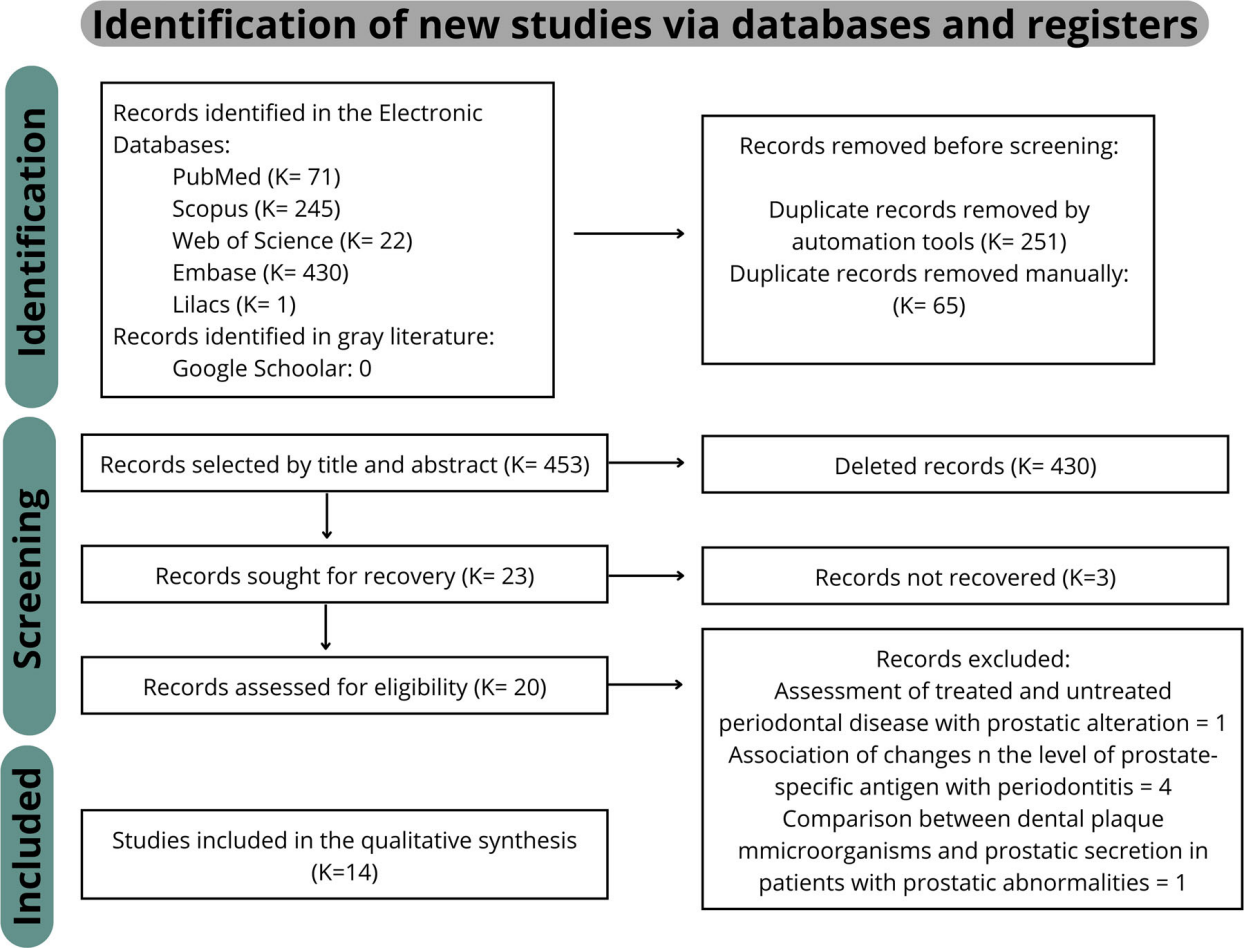
Selection of studies for systematic review through Prisma flowchart. [Color figure can be viewed at wileyonlinelibrary.com]

Initially, 769 references were identified, with 316 duplicates by automatic tools and manual review, leaving 453 studies for title and abstract reading. Then, 430 studies were excluded after this reading, leaving 23 for full reading, of these, 3 could not be recovered. Of the remaining 20 articles, 6 were excluded due to complete reading, where one investigated the presence of the same oral pathogens in samples of dental plaque and prostatic secretion in patients with prostatitis and periodontal disease [24], another four associated the levels with prostate specific antigen (PSA) with periodontits [31, 32, 33, 34]; and one evaluated the existence of an association between treated and untreated periodontal disease with prostate alteration [35], leaving 14 that were included in this systematic review. Figure 1 demonstrates the study selection process in detail.

### 3.2 Study Characteristics

Twelve of the 14 included studies are cohort studies, being six prospective [36, 37, 38, 39, 40, 41] and six retrospective studies [25, 42, 43, 44, 45, 46], and two are cross sectional [47, 48]. Adding the total number of study participants, the result was 191,680 people. The studies evaluated ages between 25 years old and 79 years old. Most of the participants were middle‐aged or elderly men, except for studies that evaluated cancers other than prostate cancer, which also included women in their research.

The studies were carried out in different countries, such as United States of America [36, 38, 39, 40], London [37]; Turkey [42, 44]; Korea [43, 45, 48]; China [47]; Taiwan [25, 46]; Germany [41].

For the diagnosis of periodontitis, one study did not specify their evaluation method [25]. Seven studies used clinical examinations to diagnose periodontitis, with the primary measurements being periodontal pocket depth, bleeding on probing, tooth mobility, and clinical attachment level [36, 40, 42, 43, 45, 47, 48]. One study [46] despite clinically evaluating, did not specify the clinical evaluations conducted. One study determined the periodontitis diagnosis simply by reviewing dental health records [41]. Two studies used radiographic parameters of periodontal disease according to the American Academy of Periodontology Periodontal Disease Classification System [42, 44], while one study diagnosed periodontal disease by radiographic evaluation without specifying which parameter was used [39]. Self‐reported tooth mobility was demonstrated in one study [38]. Three studies [37, 38, 45] evaluated tooth mobility through clinical evaluation. Two studies evaluated alveolar bone loss radiographically [37, 43]. Michaud et al. (2018) [40] evaluated the presence of gingival resection. Hujoel et al. (2003) [36] and Wu et al. (2019) [47] examined the presence of calculus and the extent of gingival inflammation.

Regarding prostatic changes analysis, ten studies evaluated medical records [25, 37, 38, 39, 40, 42, 43, 44, 45]. In one investigation, which investigated the association with benign prostatic hyperplasia (BPH), described prostate measurements and volume by ultrasonography and physical evaluation [47]. One study utilized death certification caused by prostatic cancer [46], and another used medical diagnoses of BPH and prostatitis [25]. Another research, which studied the association with Lower Urinary Tract Symptoms/BPH used uroflowmetry, transabdominal ultrasonography and transrectal ultrasonography [48], and the last study registered files using WHO ICD‐10 codes [41]. Only one paper did not specify the method for diagnosing prostatic abnormality [36].

Twelve of the fourteen articles showed association between periodontitis and prostate cancer [36, 38, 39, 42, 43, 44, 45, 46], showing a higher prevalence of this type of cancer in participants with periodontitis compared to those without periodontitis. Two studies [37, 40] showed no association and one [41] showed a trend toward a significantly difference, since the data after controlling for education, income and age showed no statistical difference between the groups with and without periodontitis.

Three studies found association between periodontitis and benign prostatic hyperplasia (BPH). Hyun et al. 2021 [48] showed a higher number of cases of BPH among participants with periodontitis compared to those without periodontitis while two studies [25, 47] showed that patients with periodontitis were more likely to be diagnosed with BPH. About the association with prostatitis, one study [25]stated that participants with periodontitis were more likely to be diagnosed with this prostatic disorder. There was association between periodontal disease and prostatic changes in most studies, only two results showed an absence of difference between the groups [37, 40]. Table 2 summarizes and presents more details of the included studies’ characteristics.

**Table 2:** Table of data extraction from the included studies.

Author (Year)/Country/Study design	Participants			Diagnosis		Results
	Source of sample	Sample size	Age	Periodontitis	Prostatic alterations	
Hujoel et al. [36]/USA/Prospective cohort study	NHANES I (National Health and Nutrition Examination Survey I)	11,328	25–74	Extent of gingival inflammation, presence of periodontal pockets, firmness of a tooth in its socket according to Russel Index	Medical examination, a standardized medical history, laboratory tests, and death certificates following the malignant neoplasms (International Classification of Disease)	Individuals with periodontitis had elevated risk of death from cancer
Michaud et al. [37]/UK/Prospective cohort study	Departament of Nutrion, Havard University School of Public Health	150	40–75	Radiographic examination, probing depth and tooth loss	Cancer diagnostic questionnaires published by the Department of Nutrition, Harvard University School of Public Health,	This study demonstrated a modest increase in cancer risk among men with periodontal disease.
Arora et al. [38]/USA/Prospective cohort study	Swedish Twin Registry	526	38–77	Self‐report teeth mobility and tooth loss	Data collection from the Swedish National Cancer Registry, clinical records and autopsy reports	The study indicated that shared underlying genetic factors may contribute to an increased risk of both periodontal disease and cancer.
Michaud et al. [39]/USA/Prospective cohort study	Departament of Nutrion, Havard University School of Public Health	696	40–75	Self‐reported history of periodontal disease and radiographic examination	Questionnaire collected from the Department of Nutrition at the Harvard School of Public Health and medical records obtained from physicians/hospitals for newly diagnosed cancers	The study showed that patients with periodontal disease had higher risk to develop cancer
Dizdar et al. [42]/Turkey/Retrospective cohort study	Hacettepe university Dentistry and Oncology hospitals	280	Median age 49, 6	Standard clinical and radiographic parameters based on the American Academy of Periodontology	Data collected from patient files and oncology records of Hacettepe University Dentistry and Oncology hospitals	The study showed increased risk of cancer among patients with severe periodontitis
Lee et al. [43]/South Korea/Retrospective cohort study	National Health Insurance Service data	934	≥ 40	Clinical evaluation of the following parameters: Bleeding on probing, probing depth, clinical attachment level, gingival inflammation and radiographic alveolar bone loss	A sample of cancer patients registered with the National Health Insurance Service (NHIS) diagnosed with prostate cancer by medical oncologists and urologists.	This study showed that patients with periodontal disease have a significantly but slightly positive in patients with prostate cancer
Michaud et al. [40]/USA/Prospective cohort study	ARIC study	317	44–66	Clinical examination of the following parameters: Probing depth and gingival recession and attachment loss	Data collection of cancer registries in Minnesota, cancer deaths certificated confirmed by of medical records and hospital discharge codes for self‐reported cases	This study showed that increasing severity of periodontal disease is associated with cancer risk
Guven et al. (2019)/Turkey/Retrospective cohort study	Hacettepe University	40	Median age: 7, 2	Clinical and radiographic parameters according with the Periodontal Disease Classification System of the American Academy of Periodontology	Data collected from patient files and oncology records of Hacettepe University Dental and Oncology Hospitals	The study showed that the presence of periodontal disease increased the risk of cancer
Wu et al. [47]/China/Cross sectional study	Henan University	2171	51.09 ± 15.25	Community Periodontal Index (CPI)	Physical examination and prostate ultrasonography	The study showed significant risk of increase benign prostate hyperplasia in patients with periodontal disease
Kim et al. [45]/South Korea/Retrospective cohort study	National Health Insurance Service–Elderly Cohort	121,240	≥ 60	Clinical examination according Korean Classification of Disease 7th revision (KCD‐7) of the following parameters: Bleeding on probing, probing depth, clinical attachment level, tooth loss	Medical history and health examination	This study demonstrated the presence of chronic periodontal disease in patients with prostate cancer.
Chung et al. [46]/Taiwan/Retrospective cohort study	Department of Health, Taipei City Government	43,052	65–74	Clinical investigation of periodontal disease performed by a dentist	Cancer death certificates according to the International Classification of Diseases	The study showed that periodontal disease can be a risk factor of prostate cancer
Fu et al. [25]/Taiwan/Retrospective cohort study	From the National Health Insurance Research Database	1824	40–79	Dentist‐provided diagnosis of chronic periodontitis according to the National Health Insurance Research Database (NHIRD)	Diagnosis provided by board‐certified urologists according to the National Health Insurance Research Database (NHIRD)	The study showed that patients with periodontal disease have a higher risk of developing prostatitis conditions
Hyun et al. [48]/South Korea/Cross‐sectional study	ot mentioned	108	52.5–58.0	Clinical examination regarding the following parameters: Bleeding on probing and probing depth	Tests on the following parameters: Prostate volume by transrectal ultrasound, measurement of post‐void residual urine volume with transabdominal ultrasound, urinary flow rate by uroflowmetry, waist circumference and prostate‐specific antigen level	The study demonstrated a close association between periodontitis and the occurrence of benign prostatic hyperplasia.
Meurman et al. [41]/Germany/Prospective cohort study	Stockholm metropolitan area	2384	62–72	Dental records providing diagnoses of periodontal disease	Data collected from the Swedish Cancer Registry, Hospital Admission Database and the Death Registry of the Epidemiology Center of the Swedish National Board of Health and Welfare	This study demonstrated the presence of periodontal disease in patients with prostate cancer.

### 3.3 Individual Risk of Bias of Eligible Studies

**Figure 2:**
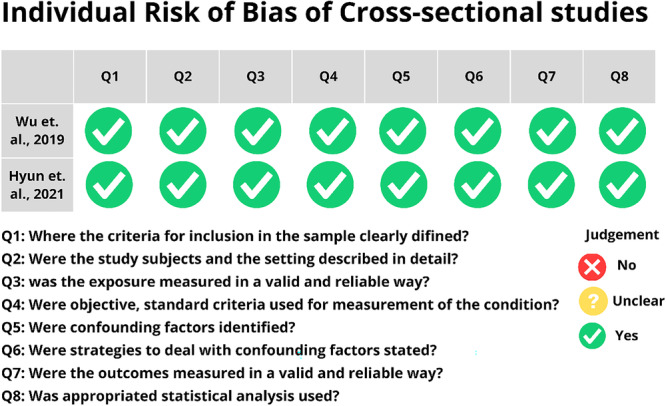
Individual risk of bias of cross‐sectional studies. [Color figure can be viewed at wileyonlinelibrary.com]

For cross‐sectional studies (Figure 2), both studies [47, 48] analyzed demonstrated good methodological quality, such as valid and reliable measurement of the result, identification of confounding factors, representativeness of the sample and use of appropriate statistical analysis.

**Figure 3:**
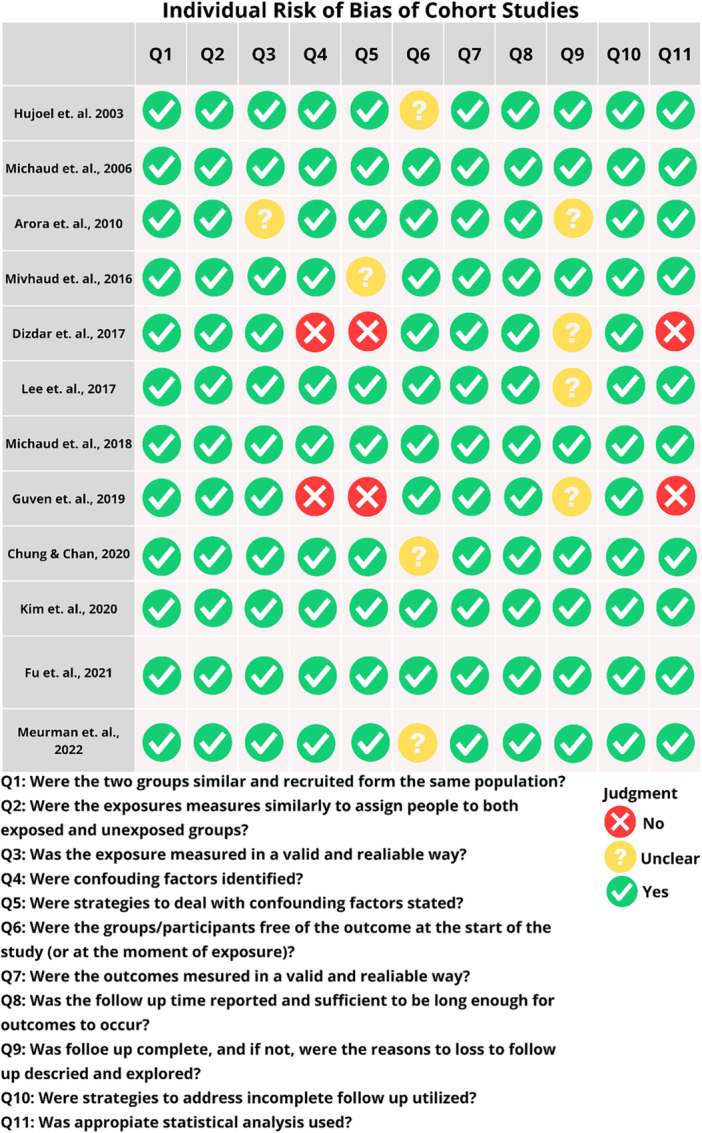
Individual risk of bias of cohort studies. [Color figure can be viewed at wileyonlinelibrary.com]

The methodological assessment of the 12 cohort studies (Figure 3) included, using the JBI Critical Appraisal Tools, revealed variations in the quality of the methods applied. In general, most of the studies performed well on questions related to the clear definition of exposure (Q1) and outcomes (Q4), with articles such as Michaud 2008 [37] and Kim 2020 [45] standing out as having obtained a “Yes” in almost all the criteria evaluated, demonstrating high methodological robustness. In addition, aspects such as statistical analysis (Q10) and the adequacy of follow‐up periods (Q11) were often well assessed in the highest quality studies. On the other hand, issues related to the control of confounding factors (Q9) and the follow‐up of participants (Q8) showed greater variability, being marked as “uncertain” or “no” in several articles, such as Meurman 2022 [41], Dizdar 2017 [42], and Chung 2020 [46], indicating weaknesses in these areas. Studies such as Dizdar 2017 [42] and Güven 2019 [44] showed more limited performance, with negative or uncertain responses in crucial areas such as the validation of exposure measurements (Q3) and the clarity of outcomes. Michaud 2016 [39] and Fu 2021 [25] also showed uncertainties in several criteria, particularly those related to follow‐up bias and confounding control.

### 3.4 Evaluation of Control Statements for Potential Confounders and Bias Consideration

Only two eligible studies [42, 44] had not performed a multivariate analysis, all other studies were selected to be critically appraised. Seven studies [37, 38, 40, 43, 45, 46, 48] made a specific mention to the term “confounding.” Only five studies [25, 43, 45, 46, 48] used the term “bias” in the Abstract or Discussion. Two studies [38, 48] mentioned non‐adjusted variables without presenting reasons: smoking status [41], genetic and inflammatory factors [48]. The other studies reported non‐adjusted variables as: unmeasured confounders or residual confounding [36, 40]; periodontal severity [25, 41, 43, 45]; periodontal treatment [37, 39]; tooth loss, denture use, and general dental conditions [47]; prostatic alterations severity [43, 45, 47]; smoking aspects [36, 41, 47]; alcohol intake [46, 47]; respiratory disease [46]; physical activity [25, 46]; nutritional status, family history, genetic, psychological and environmental factors [25]. Two studies [37, 43] mentioned that confounders were likely to affect their results, and other two studies [39, 47] considered it as unlikely. Three studies [39, 46, 47] did not state the need of caution for interpretating their results. Only five studies [36, 37, 38, 40, 45] included limitations regarding confounders in their conclusions.

### 3.5 Assessment of Confounding Factors

**Figure 4:**
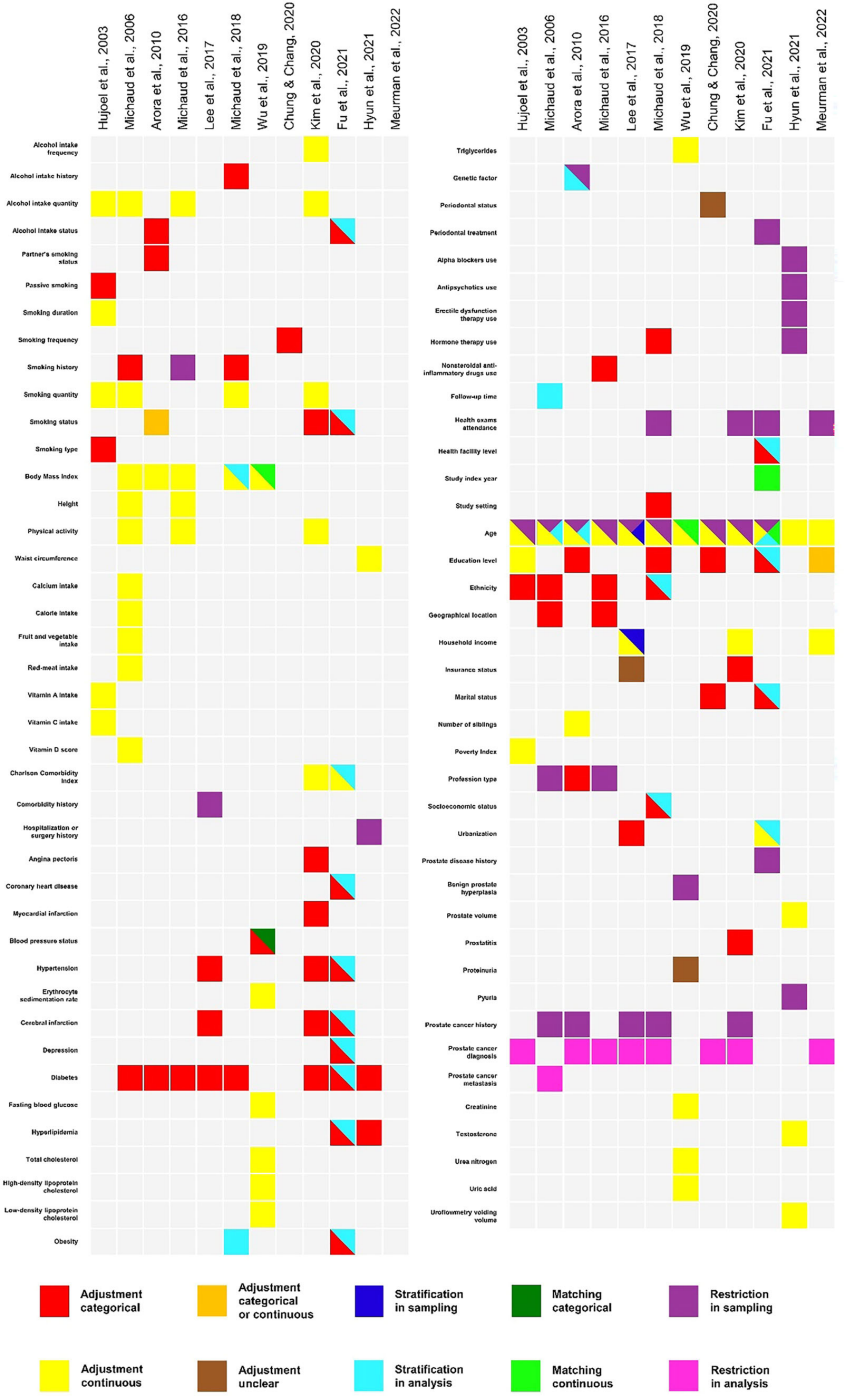
Potential confounder identified in the study. [Color figure can be viewed at wileyonlinelibrary.com]

A total of 116 different variables were identified in the selected studies, of which 81 were used in multivariate analysis to control potential confounders, showing a good proportion of adjustment (69, 83%; 81 out of 116 variables). Only one study [46] performed stratification sampling, and four studies [25, 37, 38, 40] stratification analysis. Only two studies [25, 47] performed sample matching. All studies performed restriction sampling, and only three studies [25, 47, 48] did not perform restriction analysis. Figure 4 details each potential confounder identified in the studies.

Nine confounding domains were identified in the selected studies: (1) Comorbidities; (2) Urologic Health; (3) Oral Health; (4) Body and Nutrition; (5) Sociodemographic and Socioeconomic; (6) Addictions; (7) Study Design; (8) Pharmacological Agents; and (9) Genetics. The confounding domains identified in each study are presented in Table 3.

**Table 3:** Confounding domains identified in selected studies.

Author, year	Confounding domains								
	Comorbidities (n=)	Urologic health (n=)	Oral health (n=)	Body and nutrition (n=)	Sociodemographic and socioeconomic (n=)	Addictions (n=)	Study design (n=)	Pharmacological agents (n=)	Genetics (n=)
Hujoel et al. [36]	—	x (*n* = 1)	x (*n* = 4)	x (*n* = 5)	x (*n* = 5)	x (*n* = 6)	x (*n* = 4)	—	—
Michaud et al. [37]	x (*n* = 2)	x (*n* = 3)	x (*n* = 4)	x (*n* = 10)	x (*n* = 4)	x (*n* = 4)	x (*n* = 3)	x (*n* = 2)	—
Arora et al. [38]	x (*n* = 1)	x (*n* = 2)	x (*n *= 2)	x (*n* = 1)	x (*n* = 5)	x (*n *= 4)	x (*n* = 3)	—	x (*n* = 1)
Michaud et al. [39]	x (*n* = 3)	x (*n *= 1)	x (*n *= 3)	x (*n *= 6)	x (*n *= 4)	x (*n* = 2)	x (*n* = 4)	x (*n* = 1)	—
Lee et al. [43]	x (*n* = 6)	x (*n *= 2)	x (*n *= 7)	x (*n *= 1)	x (*n *= 4)	x (*n *= 4)	x (*n* = 1)	—	—
Michaud et al. [40]	x (*n* = 2)	x (*n *= 2)	x (*n *= 7)	x (*n *= 1)	x (*n *= 4)	x (*n* = 3)	x (*n* = 5)	x (*n* = 1)	—
Wu et al. [47]	x (*n* = 7)	x (*n* = 6)	x (*n *= 4)	x (*n* = 1)	x (*n* = 1)	—	—		—
Chung and Chan [46]	x (*n* = 1)	x (*n* = 1)	x (*n* = 2)	x (*n* = 1)	x (*n* = 3)	x (*n* = 1)	x (*n* = 4)	—	—
Kim et al. [45]	x (*n *= 6)	x (*n* = 3)	x (*n* = 6)	x (*n* = 1)	x (*n* = 3)	x (*n* = 4)	x (*n* = 2)	—	—
Fu et al. [25]	x (*n* = 8)	x (*n* = 3)	x (*n* = 2)	—	x (*n* = 4)	x (*n* = 2)	x (*n* = 7)	—	—
Hyun et al. [48]	x (*n* = 4)	x (*n* = 7)	x (*n* = 6)	x (*n* = 1)	x (*n *= 1)	—	—	x (*n* = 4)	—
Meurman et al. [41]	—	x (*n* = 1)	x (*n* = 2)	—	x (*n* = 5)	—	x (*n* = 2)	—	—

Comorbidities domain was the most explored with a total of 20 variables, whereas Genetics domain was the least explored with only one variable. Their brief description and examples of variables identified in each of them are shown in Table 4.

**Table 4:** Description of confounding domains identified in selected studies.

Confounding domain (total varibles identified)		Description	Examples identified in selected studies
1	Comorbidities (*n *= 20)	General health parameters, systemic conditions and diseases.	Charlson Comorbidity Index, comorbidity history, hospitalization or surgery history, cholecystectomy, angina pectoris, coronary heart disease, myocardial infarction, blood pressure status, hypertension, erytrocyte sedimentation rate, cerebral infarction, depression, diabetes, fasting blood glucose, hyperlipidemia, total cholesterol, high‐density lipoprotein cholesterol, low‐density lipoprotein cholesterol, obesity, and triglycerides.
2	Urologic health (*n* = 16)	Urologic health parameters related to general, cancer‐specific, and laboratory exams alterations.	**General:** International Prostate Symptom Score, prostate disease history, benign prostate hyperplasia, prostate volume, prostatitis, proteinuria, and pyuria. **Cancer‐specific:** prostate cancer history, diagnosis, and metastasis. **Laboratory exams:** Prostate‐specific antigen, creatinine, testosterone, urea nitrogen, uric acid, uroflowmetry voiding volume.
3	Oral health (*n *= 14)	Oral health parameters related to the diseases, severity, diagnosis criteria, and treatments.	Alveolar bone loss, bleeding on probing, clinical attachment loss, dental calculus, gingival status, number of teeth, periodontal severity, periodontal status, periodontal treatment, periodontitis’ diagnosis criteria, Plaque Index, probing depth, tooth loss, and tooth mobility.
4	Body and nutrition (*n* = 14)	Body measures, lifestyle habits, and daily nutrition related to urologic and oral health.	**Body measures:** Body Mass Index, height, weight, and waist circumference. **Lifestyle habits:** physical activity. **Daily nutrition:** daily intake of calcium, total calories, fruit and vegetables, red‐meat, vitamins A, C, D, and multivitamin.
5	Sociodemographic and socioeconomic (*n* = 13)	Sociodemographic and socioeconomic characteristics.	Age, education level, employment status, ethnicity, geographical location, household income, insurance status, marital status, number of siblings, Poverty Index, profession type, socioeconomic status, and urbanization.
6	Addictions (*n* = 12)	Addictions related to alterations in urologic and oral health.	**Alcohol intake:** frequency, quantity per day, quantity per year, history and status. **Smoking:** duration, frequency, quantity per day, quantity per year, product type, history and status.
7	Study design (*n* = 10)	Methodological and epidemiological variables related to the study design.	**Methodological:** follow‐up rate, follow‐up time, health exams attendance, health facility level, study index year, and study setting (field center). **Epidemiological:** all‐cause mortality, cancer‐specific mortality, prostate disease mortality, and prostate disease survival rate.
8	Pharmacological agents (*n* = 6)	Medications related to prostatic alterations therapies.	Regular use of alpha blockers, antipsychotics, aspirin, nonsteroidal anti‐inflammatory drugs, erectile dysfunction therapy and hormone therapy.
9	Genetics (*n* = 1)	Genetic and environmental factors that may influence urologic and oral health.	Arora et al. [38] were the only ones to control this confounder using a co‐twin restriction in the sampling process and data analysis.

## 4 Discussion

In this systematic review, 14 studies were included, of which 12 were cohort studies and 2 were cross‐sectional. Most cohort studies found a positive association between periodontitis and prostatic alterations. The cross‐sectional studies also supported this association, although they had limitations regarding causality. As for the methodological quality of the studies the cross‐sectional studies had problems only in the way the exposures were measured, while the cohort articles had problems mainly in the domain related to the way the exposure was measured, and left unclear the strategies for dealing with incomplete follow‐up.

Severe periodontitis is a prevalent global health issue, affecting approximately 11% of the world's population [49]. From 1990 to 2019, the global age‐standardized prevalence rate increased by 8.44%, with an estimated 1.1 billion cases in 2019 [50]. Population growth accounted for 67.9% of the increase in prevalent cases from 1990 to 2019, highlighting the urgent need for policy changes to address this global health challenge [50].

Recent studies suggest a significant association between periodontal disease and prostatic conditions. This relationship is supported by research demonstrating the presence of oral pathogens in prostatic secretions of men with chronic prostatitis or benign prostatic hyperplasia [24]. Studies included in this systematic review, such as that by Wu et al. (2019) [47], observed that the presence of periodontitis was related to elevated levels of prostate‐specific antigen (PSA), suggesting a potential association between periodontitis and prostatic alterations.

The most frequently evaluated prostatic alteration among the studies was prostate cancer. The studies by Hujoel et al. (2003) [36] and Michaud et al. (2016) [39] reported that men with periodontitis had a higher risk of developing prostate cancer, which may be directly linked to the mechanism proposed by Vitor & Cota (2023) [51] involving the systemic dissemination of periodontal pathogens, potentially causing histological changes in the prostate that could eventually progress to cancer.

The quality of the reviewed studies is fundamental for interpreting results on the association between periodontitis and prostatic alterations. In the cohort studies, minor errors were identified, such as diagnostic validity, where some participants self‐reported their oral health conditions, which could introduce bias. Additionally, there was uncertainty about whether participants were free of prostatic alterations before entering the studies, which could affect the analysis. On the other hand, major errors were observed regarding the identification and control of confounding factors, with studies like those by Dizdar et al. (2017) [42] and Güven et al. (2019) [44] showing shortcomings in using adequate strategies to control variables influencing both oral and prostatic health, compromising the robustness of the results. The methodological quality of the studies directly impacts the credibility of the observed associations, hindering meta‐analyzes and data synthesis.

Exploring identified confounding factors, such as genetic and pharmacological factors, is essential to understanding the complex relationship between periodontitis and prostatic alterations. Studies indicate that genetic factors may play a significant role in predisposition to prostatic conditions, such as prostate cancer [52], but only a limited number of studies, such as Arora et al. (2010) [38], adequately addressed these factors.

The scarcity of information on the severity of periodontitis and prostatic conditions in the reviewed studies limits the ability to evaluate how these variables interact and impact outcomes, making it challenging to establish a clear picture of the relationship between the two conditions. Diagnostic validity and control of confounding factors are crucial to ensure that results reflect the true relationship between periodontitis and prostatic alterations. Inaccurate diagnoses can distort the understanding of the impact of oral health on prostatic health, while the lack of rigorous control for factors like age and family history can lead to misinterpretations.

Data on the association between periodontal disease and prostatic alterations should be interpreted with caution. The confounding domains identified across the critically appraised studies were widely scratched, but not deeply explored. In general, the researchers seemed to recognize the influence of confounders, while also neglecting the data collection and further confounding analysis. For instance, genetic factors are known to play an important role in prostate cancer [53], which was the main prostatic alteration of most eligible studies, but only Arora et al. (2010) [38] performed controlling for genetic confounders. Moreover, pharmacological agents also constitute a source of confounding due to potential interactions, adverse effects, and clinical improvement of treated conditions.

Study design also holds significant impact on analyzed health outcomes, especially when considering the follow‐up time length, different study settings, healthcare facility infrastructure, and participants’ attendance to health exams. Another blind spot in the confounding domains’ exploration is the scarcity of oral and urologic health data other than the diagnosis of periodontal disease and prostatic alterations, such as their severity, treatments, and further data on other concomitant dental and urologic conditions. Lastly, cause‐and‐effect association between periodontal disease and prostatic alterations cannot be inferred based on the eligible studies due to the study design limitations of observational studies.

The limitations of observational studies also pose a significant challenge in attempting to establish cause‐and‐effect relationships between periodontitis and prostatic alterations. These studies often rely on self‐reported data and do not adequately control for a variety of confounding factors, which can lead to misleading conclusions. The observational nature of these studies prevents causal inference, as it is not possible to determine whether periodontitis truly contributes to prostatic alterations or if both conditions are influenced by common external factors. Thus, the lack of robust data on the severity of conditions and the inadequacy of study designs complicates the understanding of interactions between oral and prostatic health, highlighting the need for future research that addresses these gaps more rigorously.

## 5 Conclusion

This systematic review shows a potential association between periodontitis and prostatic alterations, particularly prostate cancer, benign prostatic hyperplasia (BPH) and chronic prostatitis. Most of the studies analyzed indicated an increased risk of prostatic alterations in individuals with periodontitis, suggesting an association between the comorbidities. Although the analyzes point to a significant relationship, especially in cohort studies, the presence of poorly controlled confounding variables and the lack of standardization in assessing the severity of the conditions limit the robustness of the evidence. Even so, the findings highlight the impact of oral health on systemic health, reinforcing the importance of preventive interventions in both areas.

However, methodological heterogeneity and the reliance on self‐reported data in some studies make it difficult to draw definitive conclusions about the association between the periodontitis and prostatic alterations. In addition, in the analysis of the quality of bias, important errors were pointed out, especially in cohort studies, which makes it even more difficult to reach a concise conclusion. Prospective studies, with adequate control of confounding variables and standardized measures, will be essential to validate and elucidate the mechanisms of this possible interrelationship, strengthening clinical understanding and integrated management strategies for these conditions.

## Conflicts of Interest

The authors declare no conflicts of interest.

## Supporting information


**Supplementary Material:** Search strategy.

## Data Availability

The authors that the data supporting the findings of this study are available within the article and its supplementary material. Raw data are available from the corresponding author, upon reasonable request.
