# Supplementary material for: Is There Any Association Between Periodontitis and Prostatic Alterations? A Systematic Review
Source: Prostate. 2025 Aug 10;85(15):1369–85. doi: 10.1002/pros.70029 (PMC12452812; doi:10.1002/pros.70029)
Supplement: Supplementary file 1 — Supplementary Material: Search strategy. [file PROS-85-1369-s001.docx]

**Supplementary Material: Search strategy.**

Is periodontitis associated with prostate alteration?

P - Men

E - Presence of prostate alterations

C - Absence of prostate alterations (prostate cancer, prostate hyperplasia, prostatitis…)

O- Periodontitis

Databases: Pubmed, Web of Science, Embase, Lilacs, Scopus

Gray literature: Google schoolar

|  | **MESH** | **KEYWORDS/ENTRY TERMS** |
| --- | --- | --- |
| **P** | **Humans** | Man, Modern  Modern Man  Modern Man  Homo sapiens  Human |
|  | **Adult** | Adults |
| **E** | **Prostatic Diseases** | Prostatic Disease  Disease, Prostatic  Diseases, Prostatic |
|  | **Prostatic Hyperplasia** | Adenoma, Prostatic  Adenomas, Prostatic  Benign Prostatic Hyperplasia  Benign Prostatic Hyperplasias  Benign Prostatic Hypertrophy  Hyperplasia, Benign Prostatic  Hyperplasias, Benign Prostatic  Hyperplasia, Prostatic  Hypertrophies, Prostatic  Hypertrophy, Benign Prostatic  Hypertrophy, Prostatic  Prostatic Adenoma  Prostatic Adenomas  Prostatic Hyperplasia, Benign  Prostatic Hyperplasias, Benign  Prostatic Hypertrophy  Prostatic Hypertrophies  Prostatic Hypertrophy, Benign |
|  | “Prostatic Neoplasms” | Prostatic Cancer  Prostatic Cancers  Cancer of Prostate  Cancer of the Prostate  Cancer, Prostate  Cancer, Prostatic  Cancers, Prostate  Cancers, Prostatic  Prostate Cancer  Prostate Cancers  Prostate Neoplasm  Prostate Neoplasms  Prostatic Neoplasm  Neoplasm, Prostate  Neoplasm, Prostatic  Neoplasms, Prostate  Neoplasms, Prostatic |
|  | Prostatitis | Prostatitides  Acute Bacterial Prostatitides  Acute Bacterial Prostatitis  Asymptomatic Inflammatory  Bacterial Prostatitides, Acute  Bacterial Prostatitides, Chronic  Prostatitides, Chronic Bacterial  Chronic Bacterial Prostatitides  Bacterial Prostatitis, Acute  Bacterial Prostatitis, Chronic  Chronic Bacterial Prostatitis  Chronic Prostatitis with Chronic Pelvic Pain Syndrome  Inflammatory Prostatitis, asymptomatic |
| **O** | **Periodontitis** | Periodontitides  Pericementitis  Pericementitides |
|  | **Chronic Periodontitis** | Chronic Periodontitides  Periodontitides, Chronic  Periodontitis, Chronic  Periodontitis, Adult  Adult Periodontitis  Adult Periodontitides  Periodontitides, Adult |
|  | **Periodontal Diseases** | Disease, Periodontal  Diseases, Periodontal  Periodontal Disease  Parodontosis  Parodontoses  Pyorrhea Alveolaris |
|  | **Periodontal Atrophy** | Periodontal Atrophies  Atrophy of Periodontium  Periodontium Atrophies  Periodontium Atrophy  Gingivo-Osseous Atrophy  Gingivo Osseous Atrophy  Gingivo-Osseous Atrophies |
|  | **Alveolar Bone Loss** | Alveolar Bone Losses  Alveolar Process Atrophy  Alveolar Process Atrophies  Alveolar Resorption  Alveolar Resorptions  Resorption, Alveolar  Resorptions, Alveolar  Bone Loss, Periodontal  Bone Losses, Periodontal  Periodontal Bone Losses  Periodontal Bone Loss  Periodontal Resorption  Periodontal Resorptions  Resorption, Periodontal  Alveolar Bone Atrophy  Alveolar Bone Atrophies  Bone Atrophies, Alveolar  Bone Atrophy, Alveolar  Bone Loss, Alveolar |
|  | **Periodontal Attachment Loss** | Attachment Loss, Periodontal  Loss, Periodontal Attachment |
|  | **Periapical Periodontitis** | Periapical Periodontitides  Periodontitides, Periapical  Periodontitis, Periapical  Periodontitis, Apical  Apical Periodontitides  Apical Periodontitis  Periodontitides, Apical  Periodontitis, Acute Nonsuppurative  Acute Nonsuppurative Periodontitides  Acute Nonsuppurative Periodontitis  Nonsuppurative Periodontitides, Acute  Nonsuppurative Periodontitis, Acute  Periodontitides, Acute Nonsuppurative |
